# Methodological insights from the inside: developing autistic-friendly interviewing in a group interview space

**DOI:** 10.3389/fpsyt.2025.1659580

**Published:** 2026-01-27

**Authors:** Åsa Hedlund, Maria Eriksson Wester, Pia Edenvik, Cecilia Ingard, Kajsa Isakson, Lisa Kron Sabel, Danielle Uneus, Hanna Bertilsdotter Rosqvist

**Affiliations:** 1 Avdelningen for Vardvetenskap, Hogskolan i Gavle, Gävle, Sweden; 2 Independent Researcher, Stockholm, Sweden; 3 Avdelningen for Socialt Arbete Kriminologi Och Folkhalsovetenskap, Hogskolan i Gavle, Gävle, Sweden; 4 Disciplinary Domain of Humanities and Social Sciences, Faculty of Arts, Department of Game Design, Uppsala University, Uppsala, Sweden; 5 Institutionen for Sociala och Psykologiska Studier, Karlstads Universitet, Karlstad, Sweden

**Keywords:** autistic cognition, autistic forms of sociality, autistic processing styles, autistic-friendly interviewing, communication styles, cross-neurotype communication, epistemic healing space, group interview space

## Abstract

**Background:**

A neurodiversity-informed approach recognizes the perspectives and experiences of autistic people from an insider perspective, yet little attention has been given to adapting qualitative methods to account for the impact of cognition, sociality, and communication style of both interviewers and participants. The aim of this paper was to contribute to the development of autistic-friendly interviewing in a group interview space.

**Methods:**

The paper is a collective reflexive pilot study, exploring experiences of autistic interviewer and autistic participants in group interviews. The method followed five steps, from deciding what type of interview questions to use to test them in the group interviews. An autistic interviewer conducted three group interviews, each with two to three autistic participants. Interviews and analyses were conducted in Swedish, and the data were then translated into English by the authors.

**Results:**

The study identified key insights around four areas: participants’ mixed views on the interview guide, the need to accommodate autistic processing styles, the importance of recognizing autistic forms of sociality, and the significance of conducting interviews within an autistic-friendly space that fosters comfort and understanding.

**Conclusion:**

The paper outlines step by step the procedure of conducting group interviews with autistic people. We illustrate ways to capture the possibilities of autistic-friendly interviewing by working with—rather than against—autistic cognition, sociality, and communication styles in interviews with autistic people.

## Introduction

Conducting successful interviews requires careful consideration of how participants process and express information. To obtain rich and meaningful data, we need to ensure that participants understand the question, have time to think of an answer, articulate their response, and know when and how to express it. The researcher–participant relationship is crucial. For example, interview situations can be disempowering for participants if the interviewer uses specialized language or if the participants feel they are in a subordinate position of power (1, p. 1-2). The interview setting is also important. It depends on, and needs to be adapted to, communication standards in different cultures, as well as individual differences in cognition, sociality and communication style (2). One particularly illustrative case is interviewing neurodivergent groups, such as autistic people, where conventional interview formats may fail to support equitable participation. Several studies have examined interview techniques for autistic individuals, but these rarely focus on research interviews; instead, they often address contexts such as employment interviews and lack both an insider perspective and a neurodiversity-informed approach (3–5), which means that autistic participants may feel stressed about being judged during the interview (3).

In often used methodological literature within human sciences on qualitative interviews, there is commonly an implicit assumption about how people function—that is, that all people respond and react in a similar way to a given question or interview technique. For example, questions such as *“Describe the experience from the inside/as it were; almost like a state of mind: the feelings, the mood, the emotions, etc.”* (6, p. 502) and *“How do you feel about…?”* (7, p. 444) are considered to elicit rich and valuable responses about people’s experiences, opinions, and values. Researchers often rely on set interview methods that are applied across different participants. The questions asked, and how they are posed, are guided by the study’s subject and the available qualitative methods to choose from. They are also informed by the social dynamic between interviewer and participant. The literature describes the importance of being aware of one’s own role, influence, and interpretation as a researcher (6, p. 500-501; 8, p. 15). Less explored are the impact of the researcher’s cognition, sociality, and communication style in relation to those of the specific population(s) being studied, as well as the potential for adapting the interview situation to ensure ‘safe participation’ for both researchers and participants (for examples, see 9, 10).

Traditionally, it is the researcher’s behavior, approach and training that are described as crucial for the knowledge that is acquired (e.g., 6, p. 500-501; 7, p. 21). The researcher is thus considered to be the tool that shapes knowledge, which is a fair assumption. However, without an understanding of the significance of the interaction between researcher´s and participant’s cognition, sociality and communication styles in an interview situation, the tool risks failing to perform its task optimally. Building on the emerging neurodiversity paradigm (11), new methods need to be developed to investigate subjective experiences among autistic people. “The double empathy problem” (12, p. 2) has been proposed in autism research as a way to understand communication barriers between people with different cognition, sociality and communication style. The double empathy problem describes a breakdown in mutual understanding that can happen when people with different ways of thinking and seeing the world try to communicate. It’s called a “double” problem because both people are affected — it’s not just a problem within one person. Instead, the misunderstanding happens between them, in the interaction itself. For non-autistic people, this disconnect may feel strange or unexpected, while for autistic people, it’s often a familiar and common part of social life (12, p. 3). From this perspective, communication between differently disposed social actors can be understood as cross-neurotype communication (13). In contrast, speaking within a group of people with a more similar cognition, sociality and communication style can be referred to as same-neurotype communication. As autistic individuals often mask their neurodivergence in interactions with people of other neurotypes (14), there is value in exploring an autistic-autistic interview situation; involving only an autistic interviewer and autistic participants.

In inclusive research literature, accessible research opportunities and “maximizing participation” (15) is commonly referred to when it comes to enabling participant´s participation through offering participants a flexible palette of data collection possibilities adapted to the participant´s cognition, sociality and communication style. In the context of autism research, this can be summed up by the imperative of Thom-Jones and Lowe (16) to “minimise barriers, maximise flexibility” as well as to “accept autistic participants’ personal preferences to engage.” Kaplan-Kahn et al. (17, p. 3) express a similar reasoning and suggest that autistic people should be offered different ways to participate in the interview to reduce sensory overload or facilitate cognitive processing. In digital focus group interviews, for example, they should have the option to turn off their camera or choose to type their responses when they have a thought instead of speaking.

Another barrier in interview situations is what Miranda Fricker (18) refers to as hermeneutic injustice. Fricker (18) saw that women who were victims of sexual assault had no words for that because they have no opportunities to come together and formulate that. Hermeneutical injustice occurs particularly when marginalized groups find it challenging to articulate their experiences due to a lack of available interpretive resources, leading to their voices being silenced or misrepresented within broader societal discourse. As neurocognitively marginalized people, autistic people may not have many opportunities to come together to formulate their everyday experiences of being autistic, thus may find it difficult to articulate their experiences due to a lack of available interpretive resources in the interview situation. Following Fricker, this can be referred to as hermeneutical marginalization.

Commonly research on autistic people is conducted by non-autistic researchers, who are often trained in the medical “deficit model” (where autism is viewed as a cluster of cognitive impairments, rather than as a cluster of cognitive differences in comparison with non-autistic people) (17, p. 2). This poses a significant risk of reinforce the interview situation as yet another ableist and sometimes even unsafe environment for autistic people from the very moment a research question is formulated.

Following research on autistic sociality in autistic spaces, interpretive resources may be developed when autistic people come together (19, 20, p. 173-176). We will refer to this process of developing interpretive resources as “naming the nameless so it can be thought” (c.f. 21). Autistic people have described feeling more comfortable speaking with autistic interviewers (22, p. 124). Williams et al. (23, p. 4-9) reflect upon their experiences of establishing a safe space and a model of accessible communication in an “insider-only” autistic research space, where the space enabled the researchers-participants to work with their previous experiences of stigma and barriers in research participation and develop other safer, more autistic affirmative ways of research collaboration. Similarly, in the following we will explore an “insider-only” autistic research space, but more limited to an interview situation with an autistic interviewer and autistic participants, where autistic participants together with the interviewer may work against hermeneutic injustices and develop new interpretive resources (naming) to express their experiences of situations in life. In this situation, the interview situation in itself may work as an epistemic healing space (42; see also 23), as a caring and supportive space for the participants´ reflections, in which they can together with the interviewer explore and mirror each other’s experiences in an autistic-friendly and curious way.

In this paper we explore the possibilities of an insider-only autistic research interview situation (c.f. 23, p. 3-4). In a notion of an insider-only autistic research interview situation, we include firstly, a shared understanding of autism drawn from personal lived experience, and autism theories are being developed during the encounters from at ‘least partly internal observations’ (c.f. 24) as part of an ongoing naming to express our experiences of situations in life. Secondly, as being members of what autistic theorist David Gray-Hammond (25) has defined as *neuroculture*: as a “culture created by a collection of neurocognitive identities in a shared environment”; we are all insiders of autistic communities in Sweden. We think of being central in an insider-only autistic research interview situation – where the interviewer is a skilled communicator in autistic ways of communication and has an ability to facilitate autistic communication in interviews with autistic people. This, we suggest, ensure safer participation and enable participants to explore and name their experiences (c.f. 26, p. 1419; 22, p. 124).

This paper is based on a collective reflexive pilot study which is part of a larger Swedish research project led by HBR, “Autistic Flourishing and Societal Services”. This pilot served as a preparation for one of the sub-studies in the project, which focuses on expanding the understanding of autistic flourishing; its attributes and indicators. The aim of this study was both to “pre-test” or “try out” the interview guide as well as developing the interview method and the actual execution of the interview in itself, aiming for securing an interview space adapted to the cognition, sociality and communication styles of the participants. During the study we evaluated: 1) an interview guide constructed to capture autistic flourishing from autistic people’s perspective, 2) a group interview situation designed for safer participation and supporting autistic people to together with the interviewer explore and name participants´ experiences of autistic flourishing.

## Methods

This study is a collective reflexive pilot study with a phenomenological design. The study involved two stages of data production: Stage 1, initial data collection through group interviews; and Stage 2, post-interview reflective writing. The choice of group interviews was made in agreement with one another, as we felt it was easier to know what to say when we could associate from others’ descriptions.

The group of authors have different roles in the larger project:

1. Researchers: ÅH and HBR.
2. Members of co-creation group: KI, CI, DU.
3. Members of reference group of health professionals: MEW, PE, LKS.

As ÅH has the role of the interviewer in the main study, she took the role of the interviewer in this study as well. The rest of the group participated in the interviews as if they were participants in the main study. The group includes late-identified autistic and AuDHD people, assigned female at birth. All of us are Swedish, most of us are white. We are professionally and/or socially connected in different ways – some of us know each other, and some are new acquaintances. This is the first time we have worked together as a group.

Many of us bring a clinical health care and health promotion perspective stemming from training in various health professions (nursing, medicine/anesthesia and psychology/neuropsychology), while others have a background in social work/sociology, education or art/design. We approach this work from a combination of:

- A neurodiversity approach (27).
- A bio-psycho-social perspective (28, p. 576).

Based on these approaches, we view human functioning as arising from interactions between the person’s strengths and challenges inside themselves and the facilitating and hindering factors in the environment, without neuronormative judgements of human behaviour and functioning.

We have chosen to refer to our own voices in the text with a collective “One of us”. This is a way of stressing the text as written in a collective space, the collective “I” as “One of us”. “One of us” is also used as an expression of a “joint action” which feminist researchers Francis and Hey (29, p 231) have stressed as a “core to feminist action over the years but in particular within academia” where joint action counter-narrate the position as “individual experts”. The use of a collective I, is a way to counter-narrate the image of “the sole”, individualized neurodivergent, and rather stress the presence of a neurodivergent togetherness but also to protect ourselves against epistemic violence and position ourselves– as hermeneutical marginalized people.

### Constructing the interview guide and test it in group interviews

**Figure 1:**
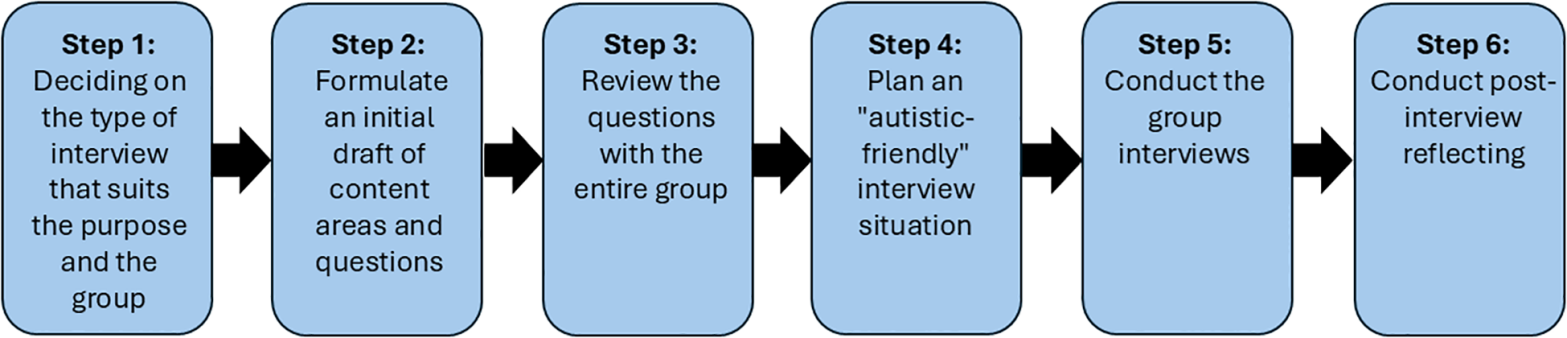
Overview of the methodological process.

To answer our research aim, we conducted a methodological process in six steps, inspired by Kallio et al.’s (30, p. 2954–2965) method for developing interview guides for qualitative research. We were also inspired by the recommendations of Nicolaidis et al. (2) regarding how interviews can be conducted with autistic people, particularly with respect to the design of interview questions and the interview situation. **Figure 1** provides an overview of the methodological process. We constructed and evaluated the interview guide following the six steps over a period of approximately two months (between the end of 2024 and beginning of 2025).

*The first step* was to determine which type of interview guide/questions would be suitable for us as an autistic group of participants to share our lived experiences. We concluded that a semi-structured interview would be appropriate since it is not entirely open-ended (we thought it might be difficult for autistic people to get started and associate if the interview topic was too vague) but also not too specific (which would not be ideal for the exploratory nature of the study—investigating autistic flourishing). A semi-structured interview guide also allows flexibility regarding exactly what is discussed and in what order the questions are addressed.

*In the second step*, we (HBR and ÅH) used our combined understandings of autism drawn from both our personal lived experience, and at least partly internal observations, us as members of autistic neurocultures and academic knowledge in the autism research field to formulate an initial draft of content areas and questions. We aimed for the questions to have a bottom-up approach (i.e., detail oriented), as this aligns with autistic cognition (31, pp 103-122). In addition to drawing on our existing knowledge, we also reviewed literature on autistic flourishing to understand what had been previously done and to gain a deeper understanding of the phenomenon and its position in the development of knowledge. Before moving on, researchers from the larger project reviewed the interview questions before we proceeded to the next step.

The *third step* involved the rest of us (KI, CI, DU, MEW, PE, LKS) reviewing the questions and providing feedback—specifically focusing on assessing their clarity and relevance, suggesting revisions to existing ones, and proposing additional questions where gaps were identified—which was then sent back to ÅH and HBR. The feedback given mainly concerned phrasing and word choice. For example, it became clear to us that certain question formulations could be interpreted in very different ways/were unclear. Suggestions were made regarding which word to use to best capture flourishing (we ultimately settled on the description “må bra” [feeling well], which we defined as “experiences of ‘feeling well’ in the body, brain, and mind. It encompasses both small satisfactions and moments when things feel at their best. These experiences can, for example, include emotions, sensations, feelings, or perceptions”). The final interview guide consisted of 18 questions, divided into six topic areas, and is presented in **Table 1**.

**Table 1:** The interview questions (originally in Swedish, translated to English for this paper).

Content area	Question
The experience of a good autistic life in general	1. How does it feel inside you when you feel good? 2. What do you usually do when you want to increase a good feeling inside yourself? 3. What in everyday life, or which moments in everyday life, usually make you feel good inside? 4. In what other moments, in life in general, does it feel good inside you?
A good autistic life in interaction with others. Interaction can mean meeting others in person, online, or by phone. It can take place in private settings or at work.	5. How would you describe how it feels inside you when you feel comfortable together with others? For example, you might feel light in your body or experience a tingling sensation. 6. In what situations with others does it feel good inside you? 7. What do you do to create a good feeling inside yourself when you are in contact with or interacting with others? 8. Many autistic people find interaction with others difficult, and many may experience negative feelings afterward, such as fatigue or overthinking. What do you do after interacting with others to feel good?
A good autistic life when you are alone	9. How does it feel when you feel good when you are alone? 10. In what situations, when you are alone, do you feel good? 11. What do you do when you are alone to feel good?
To work preventively on your own in the long term	12. Do you have any examples of how you try to take care of yourself in order to feel good in the long run?
Concluding questions: other ways to create a good autistic life	13. Is there anything else, beyond what we’ve already talked about, that contributes to you feeling well in the moment? If yes, what? 14. Is there anything else, beyond what we’ve already talked about, that contributes to you feeling well in the long term? If yes, what? 15. Set aside all obstacles and describe what your ideal life would look like — that is, a life where you feel your very best.

*The fourth step* was to plan an autistic friendly interview setting, i.e. interview settings that would be adapted to the cognition, sociality and communication style of autistic people. We concluded that flexibility and clarity were important. This is described in more detail in *the fifth step*, which was to test the interview guide in the real interview situation.

The three interviews took place in January-February 2025 using the digital conferencing tool Zoom because of geographical and energy saving reasons (we live far away from each other and travelling feels energy consuming). There was about a week between each interview. Each interview included the interviewer and two to three participants. In the first interview, HBR, MW, LKS, and ÅH participated. In the second interview, CI, KI, PE, and ÅH participated, and in the third interview, HBR, DU, and ÅH participated. ÅH led the discussion (asking questions, guiding the conversation when needed, and keeping track of time) but was also an active participant in the discussions. The fact that ÅH was the interviewer and participant in the discussion at the same time reduced the feeling of power imbalance between the interviewer and the participants. The interview developed more like a conversation between all of us who shared experiences from our own lives.

At the beginning of each interview, ÅH encouraged all of us to “act autistically” during the interview. This meant that we were explicitly encouraged to respond to our diverse range of cognitive processing needs, see examples in **Table 2**.

**Table 2:** Examples of autistic behaviors encouraged in the interview.

Taking a break when needed
Allow for silences in the interview where everybody took time to process what had been said
Make notification in a note book in order to process our own thoughts before speaking Typing instead of speaking
Ask when things feel unclear
Turning off the camera
Stimming
Moving around
Engage in parallel activities (such as playing a game on the phone or knit)

The time for the whole interview was pre-set to 1 hour. It consisted on two parts: interview following the interview guide (45 minutes) and post-interview reflection (beginning of the *sixth step*) where we were reflecting upon our experiences of being interviewed and the interview guide (15 minutes). The time limit of 1 hour was based on cognitive ability to focus and need for recovery. However, it was not enough to go through all questions, which stressed the importance of an interview situation divided into several parts with time in between allowing for post-interview processing.

The interviews were video recorded. All had our cameras on at some point during the interview; some of us the whole time and some of us parts of the time. ÅH encouraged the rest of us to share any post-interview reflections after the interview (continuation of the *sixth step*), as the discussion might have sparked thoughts that we did not have time to process and express during the interview. One of us chose to supplement their interview with a written response a few days later.

### Analysis

An inductive thematic analysis was conducted informed by Braun and Clark´s approach (8, p. 51-117). Each interview was fully transcribed word for word by Amberscript (32). ÅH then listened to the interviews and made corrections to the text. Then, HBR and ÅH reviewed the transcriptions and created the first draft of the analysis, i.e. sorted the data based on similarities and differences and created initial themes. During the analysis process, all of us analyzed the data material to let the data guide us in how to present the results (continuation of the *sixth step*). An “analysis- and writing schedule” was established by HBR in consultation with the rest of the group to ensure that everyone knew which days they would be working on the analysis and other text. This approach made the process clear and secure for everyone involved while also ensuring the quality of the analysis, as each person could add reflections as well as “correct” or clarify what they had said during the interview. Reflections and the interpretations of the data thus developed continuously during the process of collaborative writing. Through this iterative process, themes (i.e., pattern of meaning based on our lived and shared experiences) gradually emerged over time, under which we later identified subthemes. Our data analysis was grounded in a neurodiversity approach and the biopsychosocial model, meaning that we analyzed the data with neuroaffirmative values and remained attentive to the interaction between us and our environment.

All interviews and transcripts were conducted in Swedish. For the manuscript, the interview questions and selected quotes were manually translated into English by ÅH, occasionally consulting digital dictionaries or ChatGPT for word choice, and reviewed for semantic equivalence by HBR. Special attention was paid to preserving our’ language style, including expressions characteristic of autistic communication.

## Results

**Figure 2:**
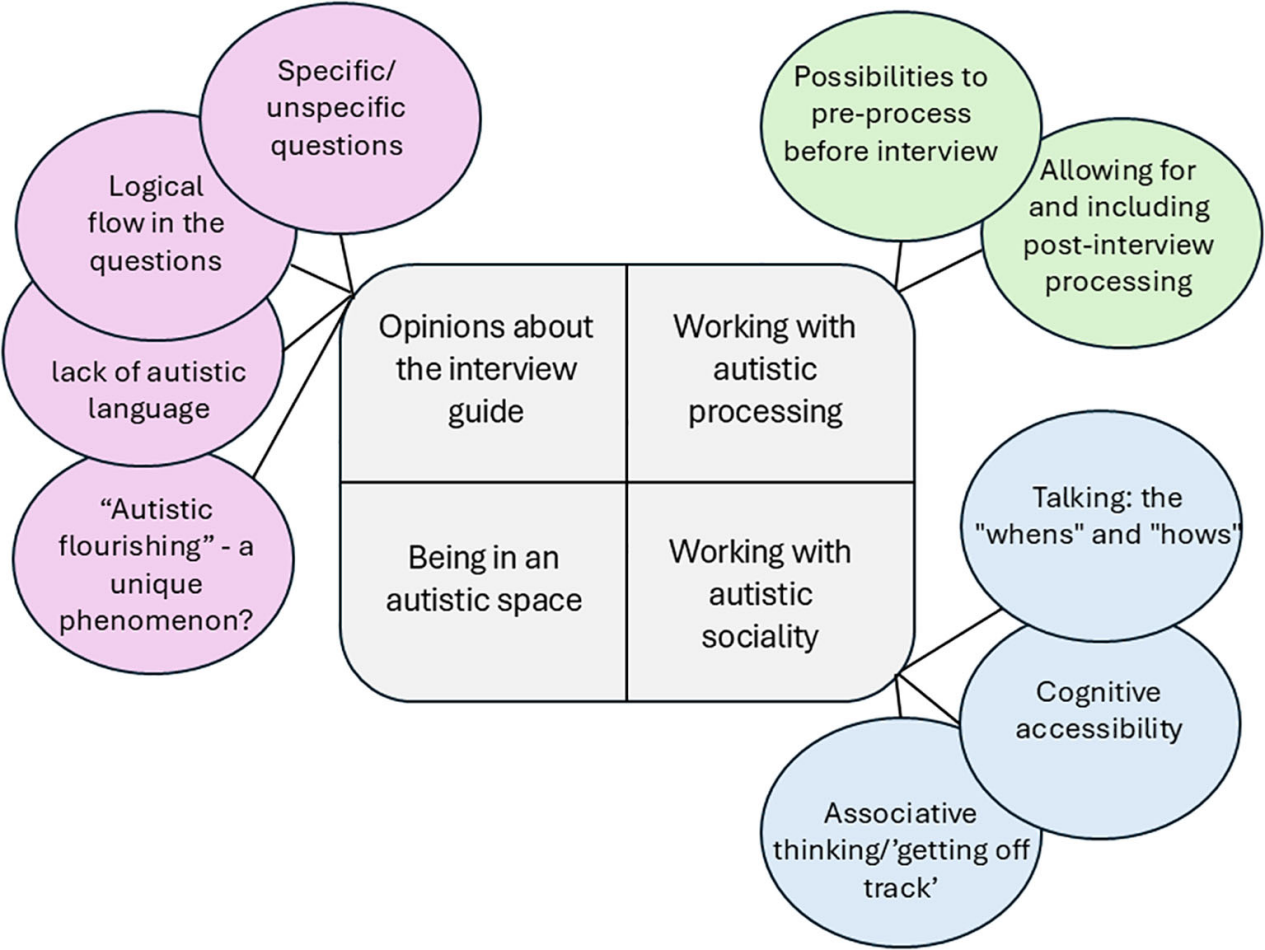
Overview of themes.

The findings present the outcome of the study’s two different goals: first, to evaluate an interview guide constructed to capture autistic flourishing from autistic people’s perspective and second, to evaluate an interview situation designed for safer participation for autistic people. The findings are presented using four themes and ten subthemes, see **Figure 2**.

### Opinions of the interview guide

#### Specific/unspecific questions

We talked about the specificity of the interview questions and agreed that they were experienced as broad/general, illustrating a phenomenon that we during the process started to name “top down questioning”, more adapted to neurotypical cognitive styles. On the one hand, we felt that this made it difficult to “get at” what we were looking for — it was easy to get stuck in generalized topics like “exercise and diet.” On the other hand, we felt that keeping the questions open was good in order not to exclude anything valuable. While we were critical of the open questions and wanted more specific phrasing, we also found it difficult to suggest concrete alternatives. One of us pointed out that overly narrow, detail-oriented more in line with a bottom-up-approach commonly associated with an autistic cognitive style, questions could threaten people’s ability to freely associate and bounce ideas off each other (which was the point of the group interview), and that we might lose what we’re looking for that way as well.

The interviewer, however, felt that she found an answer to the first question by responding to question three:

I realized that by talking about what the problem is when you’re together with others, and how you handle it, I arrived at the answer to question one — how it feels inside me when I feel good. Because these questions are so general. In the earlier interviews [interview one and two in this study], it’s also come up that it’s hard to pinpoint how it feels inside when you’re feeling good. It’s easy to land in things like: “It feels calm and nice in my body after I’ve exercised.” But I’ve been advised [in the earlier interviews] that you need to dig deeper. And then I thought — how do you dig deeper? This was, for me at least, a way of getting at how it feels when you feel good. Digging into: what’s the goal of fixing yourself? Where do I want to end up? There you go. So maybe that can be a little clue in how to phrase the question — how to help people arrive at how it feels inside them when they feel good. (the interviewer).

Several of us suggested asking for specific examples, although we also acknowledged the risk that people might feel stuck if they have to recall memories to come up with examples. One of us suggested including two different phrasings in the same question to spark associations in more participants. We also discussed that our experience of the interview guide may not only depend on the guide itself, and therefore might not be solved simply by improving it. Some experiences are part of the interview process itself — for example, the uncertainty at the beginning and the “warm-up” time it takes before one feels comfortable.

#### Logical flow in the questions

In all of the interviews, one question naturally led to the next without the interviewer needing to steer very much. The first question, about what it feels like to feel good, was difficult to talk about without moving into the next question — that is, about other people. Two of us expressed the logical flow like this:

One of us: I think the whole thing … I think the questions flowed quite dynamically.

Another one of us: I think so too. Absolutely.

#### Lack of autistic language

In exploring how autistic-friendly interviewing can capture meaningful experiences, we reflected on what flourishing might mean in autistic lives. We talked about the fact that we lack an autistic language for flourishing – naming -, which made the questions feel “neurotypically formulated”:

“But it becomes like this: How do you feel when you feel good? That feels like such a neurotypical question. What the hell is ‘good, ‘ anyway?” (one of us).

One of us described feeling angry at the word “good, “ that it feels bland and without meaningful content. We all agreed on the challenge of finding a better, more “autistic-adapted” word — one that hasn’t been so overused and isn’t strongly associated with neurotypical communication — but we became frustrated by how difficult it was to come up with alternative naming of our experiences. Some suggestions that came up were “a pulse that feels right” or “an energy that is positive.”

#### “Autistic flourishing” - a unique phenomenon?

We talked about the possibility that there might not be a specific “autistic flourishing, “ but rather that flourishing is experienced similarly by all people regardless of neurotype. However, the path to it may differ between neurotypes, as one of us expressed:

“They (neurotypicals) maybe … I might feel the same type of well-being. But I feel good from talking about something deep and really diving into it with another person … but the feeling inside us might actually be quite similar to what they (neurotypicals) feel. It’s just that the activities or the kinds of communication that create those feelings are completely different. Like, I might like to be alone and go for a run, and they might like sitting in a group drinking beer. But the actual feeling of well-being might not be so different between us. So, I don’t know if I want to — I don’t know if I’m convinced that autistic flourishing is an entirely separate feeling from neurotypical flourishing. Rather, I think the autistic path to flourishing is different. That’s how I would put it. And I don’t think I am, like, fundamentally different from a neurotypical person.” (one of us).

Following this, we emphasized that even if the content of flourishing may be similar between neurotypes, the autistic path to flourishing should not be confused with a neurotypical one — for example, it should not turn into us being encouraged to “learn” to enjoy neurotypical social situations.

When it came to the experience of a possible “autistic flourishing” as entity or phenomenon — and whether such a thing exists — the group was divided. Several of us felt that, as humans with similar biology, the feeling of “good” should ultimately be the same for all people. At the same time, there was uncertainty in the group about whether this is actually true or if we simply haven’t yet managed to fully “grasp” what autistic flourishing really is. One of us shared that since she often feels “broken in the brain” after a day with many social interactions, her experience of feeling “good” is when her brain feels like it’s holding together. However, this might perhaps be more about the internal metaphor or image of that experience (centered, focused, calm) being different, rather than the core feeling itself.

### Working with neurodivergent processing

#### Possibilities to pre-process before interview

As all of us had been involved in the construction of the interview guide, this mirrored receiving the interview guide in advance. It could be experienced as both helpful and hindering. It was helpful in allowing one to prepare and think about how to respond, but at the same time, it could feel like a limitation — having already formulated an answer in your head and becoming “stuck” in that. When arriving at the interview, the intention was then to deliver the answer you had already prepared, instead of feeling open and spontaneous together with the group. One of us described it like this:

“I can feel that it’s both helpful and not helpful for me to get the questions beforehand. Like, I get the chance to think and reflect and write things down in advance. But it can also make me lock myself into, like, ‘these are the experiences I want to talk about’. And once I’m here with all of you, it’s like … well, my brain doesn’t really open up to think more, but rather, ‘now I just want to say this, and now I’ve said it’. So (small laugh) um…” (one of us).

Since we had different views on this, we discussed that sending out the interview guide in advance should be an offer rather than something that is routinely done with everyone. Alternatively, that everyone could receive the questions before the first interview session, but not for the following sessions.

#### Allowing for and including post-interview processing

Since interviews may spark thoughts that one doesn’t have time to express and process during the interview, we talked about the importance of having the opportunity to follow up with the interviewer (and possibly the rest of the group) afterwards, either verbally (with/to the interviewer) or in writing (to the interviewer or the whole group). One of us suggested that it might be good to set a time window for this:

“Big or small things — but still within a set time frame. So, I don’t keep going on forever, but rather, it’s something that works for me. I might say that tomorrow is a really good day for doing that, longer time means I’ve forgotten what happened. That gives space for post-processing.” (one of us).

Several of us mentioned that having a time limit for the interview felt reassuring. Even if we didn’t manage to answer all questions or say everything we may have wanted, a lengthier interview would mean a less safe participation due to risk of cognitive overload. One of us suggested the alternative that those who still have energy could continue the interview after a 15-minute break. The suggestion was to have a 45-minute interview, a 15-minute break, and then continue another 45 minutes for those who felt for it. The break would ensure some processing time and enable participants to return to the second session either with reflections from the first session — or with new thoughts.

At the end of each interview we talked about whether we felt a need for a follow-up interview to continue the conversations. However, we realized we couldn´t know this right away. After all interviews had been conducted, we arrived at not doing more interviews but encouraged everyone to add their reflective thoughts during the process of co-writing. The following dialogue about whether or not we should have more interviews took place during the first pilot interview:

One of us: Maybe we can let it sink in a bit. Because I’m thinking, it’s also kind of fun. Right now I feel so full (small laugh) that I can hardly think of anything other than that it was fun. But I also think it might be like this — in two parts. First this trial interview, testing it out, but then also writing about it. And then it could happen that when we start writing, we realize that maybe we need another session, just because we need more data on something.

The interviewer: That sounds wise — to let it sink in a bit. That actually feels good. But I have to say, what an educational hour in so many ways. Not just methodologically, learning how to do interviews, but also how you’ve described things and helped me understand myself a bit better. So, thank you very much!

### Working with neurodivergent sociality

#### Talking: the “whens” and “hows”

As autistic people we have experiences of being perceived as “talking too much or too little”. This is commonly described in autism literature as difficulties in turn-taking in the conversation. This anxiety, of being unsure about “how much” including how detailed one´s responses should be (c.f. ‘infodumping’), and “when” one should talk was brought up as a challenge in the focus group setting- This can be referred to as part of a broader umbrella of “research anxiety” (33). One of us said:

“Then I also find it difficult in a group, and to know when. When could I jump in? And if I’ve spoken, have I said a bit too much now … and finding that balance is hard, I think.” (One of us).

The rest of us agreed with this. This is contrasted with a situation where one is only in conversation with the interviewer (individual interviews) — a situation with less cognitive load; less cognitive input, less multi-modal sensory and social complexity, and a different expectation in the turn-taking, including being expected to talk “a lot.” One of us pointed out that although individual interviews do provide more clarity about when to speak, they come with other disadvantages instead.

“Even if I were in a situation with just one interviewer, then I would become much more talkative. I would say a lot more. But I would also feel uncomfortable about saying so much.”(One of us).

During the first interview, it was therefore suggested that in upcoming group interviews (in the main research project), the interviewer should direct questions to specific participants — “go around the group” with each question — in order to avoid uncertainty about who should speak. In the second interview, this was done, and the interviewer experienced it as helpful for everyone at the beginning. However, once the conversation got going, it instead became a barrier in communication. When someone started to speak, they stopped themselves because the interviewer hadn’t directed the question to them, leading to an unnatural interruption in the interview situation. We talked about how it’s important to agree within the group on when and how it’s okay to jump in or interrupt — and that interrupting doesn’t necessarily have to be a negative thing.

“Yeah, but that’s also something you might wonder about … How is it, interrupting? What’s everyone’s relationship to that in the room? I often experience — at least in many cases with people who have ADHD — that interrupting is a way of showing you’re engaged, that you’re … it’s a way of being an active listener. But then, for others, it can be really disorienting to be interrupted. (One of us).

#### Cognitive accessibility: allowing/enabling for different processing time in synchronous data collection

In the following we explore synchronous (such as real-time interviews) versus asynchronous (such as email interviewing or written interviews that allow for different cognitive processing times, c.f. 10, 34) data collection. We talked about how it’s challenging to come up with what you want to say during the (synchronous) interview time, and that the answers you give can end up being rather short or general because we weren’t “done thinking.” Questions during an interview may trigger a lot of thoughts that grow and take shape in one´s mind during the interview. However, they may not be ready to be expressed until at the end of the interview - but then, there’s no time left. One of us expressed it like this:

It feels like it takes a while, I think, before you kind of … get into some sort of flow. In the beginning, it can be that I don’t really know what to say, and then more thoughts start to come. Then it’s time to end the interview. At that point, I could have said a lot.” (one of us).

Similarly, another of us said that she may expresses herself briefly before she has had time to fully process her thoughts, and then at the end of the interview, she may feel that there was much more she would have liked to say in relation to what the others had said.

“There’s so incredibly much more going on inside than what comes out expressively.” (one of us).

Another one of us expressed the opposite — that she was able to quickly formulate her thoughts and then became tired.

At the start, it’s usually just me (as an example), with AuDHD, doing all the talking. Then, toward the end, everyone else starts connecting and building on each other’s thoughts — but by that time, I’m already exhausted and out of energy … Sometimes my clearest thoughts come right at the beginning, and after that, my energy drops — but that doesn’t make what I said any less valuable.

At the beginning of the interviews, the interviewer mentioned the option to write in the chat if one wanted to, but the chat was never used during the pilot interviews. Several of us suggested that it would be better if the interviewer were more concrete in their instructions about when and how the chat could be used; for example, ‘when you get an association while someone else is talking and are afraid you’ll forget it or won’t have time to say it’.

#### Associative thinking/’getting off track’

We talked about the challenge of, on the one hand, daring to associate freely and, on the other hand, staying on topic. We felt that it’s important for participants to feel free to make associations and express themselves without fear that someone else will perceive it as wrong or ‘off track’. Even though the interview focuses on a certain topic (in this case, autistic flourishing), sometimes you need to take detours to get to what you really want to say. We experienced that our mainly detail-oriented, bottom-up cognitive style involved telling a story step by step, where meaning is built gradually and the point is reached through the whole sequence. We discussed how it’s important that the interviewer guides the conversation back to the topic if we go ‘off track’, but not too abruptly, allowing for this step by step-storying. We talked about how it’s important for the interviewer to be clear at the beginning of the interview that there are no right or wrong answers, that the “range of relevance” is very broad, and that free associations are encouraged.

“… to hear that free associations are completely okay. That it’s totally fine to end up here even if that wasn’t the intention. Then it just becomes important information — yes.” (one of us).

One of us described that the group interview felt comfortable precisely because it reduced the pressure to constantly have something to say oneself, and allowed time to process and make associations in your head while others were talking. Several of us also described that it was important to have the possibility to say “pass” if you needed more processing time, and that this option was introduced at the beginning of the interview by the interviewer.

### Being in an autistic space

To be in an autistic space seemed to fill different functions. At times, we drifted away from the actual interview questions and began reflecting on autistic identity and experiences of being autistic. We talked about our differences and similarities, including how we experience ourselves as different from neurotypicals, for example when it comes to interests, motivations or things that drive us forward in life. We found the conversation stimulating and had difficulty stopping, stressing the need of the interviewer as keeper of the in advance decided upon time limit. We also found common ground in how we perceive neurotypicals’ posts on social media. The group became giggly when we shared the same confusion about why neurotypicals post pictures of their food, their children, their family, and their pets. Several times, we ended up discussing things that make us, as autistic people, feel bad — even though the interview was supposed to focus on the opposite: what makes autistic people feel good. We ourselves have extensive experience of feeling bad, and we often hear about the struggles of other autistic people, which makes this a very pressing topic to talk about and address. It was difficult for us to stick only to the “positive” or the main topic of the interview. Overall, we felt we could relax more in the interview when it came to social expectations. As one of us put it:

“For me, it was important to have an explicitly autistic context, because I assume I won’t be routinely misinterpreted there.” Even though we are different, we share experiences of being different from the majority and of reflecting on that. I don’t have to be as socially guarded, which likely results in richer interview data”. (One of us).

## Discussion

The process of conducting this study provided us with several insights into how an interview guide should be designed and how qualitative interview data should be collected among autistic people to ensure safer participation and enable autistic participants to explore and develop understandings of somewhat abstract (or ‘top down’) concepts and intimate topics such as autistic flourishing. From a neurodiversity perspective, we recognize neurocognitive differences as natural variations rather than deficits. We found that being able to mirror each other in our way of functioning was helpful for the group discussion. Furthermore, coming together with other autistic people helped us to develop new words and languages (to name) that better reflected our experiences. The act of autistic-friendly interviewing became in itself a way of working against hermeneutic injustice commonly experienced by autistic people.

### Challenges and how to overcome them in autistic group interviewing

#### Working with literal thinking

As autistic people we have a tendency to consider questions very exactly and literally (35, p. 1134), also referred to as manifest communication. Our frustration regarding the lack of autistic languages (or pre-dominantly bottom-up naming) can be linked to this, as neurotypical language (or pre-dominantly top-down naming) is not suited for manifest communication. Our tendency to interpret things literally may have something to do with the idea that questions have one correct answer and that the “truth” is a valuable thing per se, and a sense that it is very important to get it precisely right. A problem may therefore present itself when the question in itself is difficult to understand, due to ambiguities of pre-dominantly top-down naming or if the fundamental problem underlying the question is unclear or hidden. “How do you feel?” might make autistic people think, “Why are you asking me this? Why is this important? Is it important at all? Or is it just a polite question with no deeper meaning?” The response will then depend on what the autistic person believes the purpose of the question is. Similar experiences were also described by autistic people in Finn et al.’s study (36, p. 2091), who found job interviews too challenging when the questions were not clear enough. This concept of taking questions very seriously and literally becomes important in the setting of research interviews. We believe that it is important not only to formulate the questions in a specific way but also to clarify the intention behind the questions and allowing for bottom-up step-by-step answering.

Another issue related to literal thinking and manifest communication is that there are so many levels on which to answer a question. Questions such as “what is a good life for an autistic person” or “How do you experience yourself having a good life - if you would describe it as sensations in your body?” may not lack clarity but they may be perceived as unclear regarding which theoretical level is being referred to because they are very broad (in line with a top down processing style). In line with a more detail-oriented, bottom up processing style, we may ask ourselves: “On what level should we answer? Why is this question important? What will it explain? Since autistic people often strive to answer literally we believe it is extra important to clarify at some point exactly on what level(s) of (top down) abstraction the study is aiming to explore. If the scope of research is very broad, it is important that researchers are clear about this as well. Questions that seem to hide a deeper meaning that we cannot understand may make us reluctant to answer and we tend to get stuck on definitions of words. However, when we understand what we really are “getting at”, or the concept of something, it is easier for us to relax and let go of the minuscule details and paint a more interesting greater picture. We therefore believe that, for autistic people to be able to share their experiences in an authentic way, it is fundamentally important to clarify the goal of the study, as this can help in understanding the questions despite different relationships with the individual words in the interview guide.

#### Getting to the core of a phenomenon

Hermeneutical marginalization of autistic people means that they not only struggle to describe their experiences but also to understand them themselves (37, p. 564). To investigate our own minds and bodily experiences, is difficult and intimidating. We may also have a strong urge of wanting to get to the core, the very essence, of things we investigate (including when we are being interviewed). We may be looking for something that could be unique to “us”, this group of people, who interpret ourselves as divergent from non-autistic people. Divergent in relation to people who we by definition can know only from secondhand account. How do we know in what ways we differ and in what ways we do not? Comparing oneself to someone whose internal perspective one has never experienced is a difficult, if not impossible, task. We can compare ourselves to the observed behaviors we see in non-autistic people, but we never gain full insight into the core that underlies these behaviors. We can take part in non-autistic people’ descriptions of how they function, but even then, we do not fully grasp it, as we may not always recognize the way they use words or the experiential sphere from which their descriptions originate (37, p. 566). The risks of framing responses based on comparisons with non-autistic functioning are that we may find it harder to articulate our thoughts (becoming uncertain about whether they overlap too much with non-autistic functioning) or that we may express them in an overly extreme way to clearly distinguish the autistic experience from the non-autistic one. This means that autistic voices may not be adequately represented. We also risk falling out of the neurodiversity approach and into the biomedical deficit perspective (38), which argues that neurotypicality is the human benchmark against which other neurotypes should be measured. We therefore suggest that, in interviews with autistic people, the focus should be shifted away from contrasting autism with neurotypicality and instead encourage autistic bottom-up exploration in a safer interview situation. The interviewer should inform participants that the study focuses on autistic perspectives, regardless of whether they overlap with a neurotypical perspective or not. The uniqueness of autistic thinking and perception will emerge naturally.

Furthermore, since we feel that we lack an autistic language, it may be valuable in future interviews to conduct more multifaceted interviews — focusing on communication through multiple forms of expression and documenting the full experience: not just spoken or written language, but also visual, sensory, and nonverbal ways of expressing oneself.

#### Adapting the setting of the interview

From a biopsychosocial perspective, we recognize that human functioning emerges from the interaction between personal, social, and environmental factors. In our case, we were discussing in groups of people (social) where most of us did not know each other in advance via a digital platform (environmental) online. This is practical and convenient (e.g., no travelling, easy to find a time when everyone can join and people can choose a place where they feel comfortable) (39, p. 1297), but it may not be the most liberating way of exploring our minds. It is known that online interviews come with certain risks, such as a sense of feeling restrained by the limited way people express themselves non-verbally during online interviews, issues with turn-taking and taking initiative to speak, and difficulties in speaking about personal matters (40, p. 6-9). In the present study, we noted a tendency to repeat and acknowledge what someone had already said, which could be due to the interview being conducted digitally, but could also be because we did not know each other and were therefore uncertain in the interaction. When people don’t know each other and the context is new, the interview can be perceived as “artificial”, which can reduce engagement during the interview (41, p. 4-5). Thus, there may have been a clash between our personal needs, other people, and the environment we were in. Regardless of the practicality of the digital setting, we wonder what it would have been like to dig deeper into the material of our collective mind in a physical meeting. To compensate for the limitations of digital platforms, we suggest conducting repeated digital group interviews. This could help participants become more comfortable with each other, the interviewer and the interview situation. The artificial interview situation becomes more of a natural social context as the participants, like other social groups, build relationships and a shared foundation to rely on. This enables deeper and more meaningful discussions (41, p. 5). To accommodate different cognitive styles and processing needs (some have a faster processing style, speak and get tired early, while others need more time to think before speaking), it can help to use a conversation format that allows for more variation. For example, starting with a bit of silence before people speak or working in pairs first can enable different cognitive styles and processing needs. It can likely have a positive effect on participants’ ability to share their thoughts when participation isn’t expected to be constant or to take a specific form.

The number of participants is also important to consider. As an interviewer, ÅH found that the optimal group size was three participants and herself/the interviewer. This group size kept the discussion lively without placing too much speaking expectations on anyone, while also meeting ÅH´s own processing needs, and enabled her to keep track of who said what and when. If the groups had been larger, it might have been difficult to fully “take in” the entire group, with attention potentially focusing on the speaker and missing what others are experiencing. We also felt that this number was optimal from the participants’ perspective, as it limits the amount of information they need to process.

#### Adapting the timeframe

The interview duration (45 minutes) were maybe too short. Several of us felt that we could have discussed longer. On the other hand, some of us appreciated that the time was not “too long” (so they didn’t get a cognitive overload). As a comparison, the non-autistic participants in Gray et al.’s study (39, p. 1295) felt that Zoom interviews should not last longer than an hour, as they similarly otherwise get a cognitive overload. Whether the time was sufficient in the present study may depend on the number of participants in the interview. The time did not feel as limited in the third interview, where there were two participants and the interviewer, compared to the first and second interviews, where there were three participants and the interviewer. Overall, however, our feeling was that the interviews did not manage to capture everything that should have been captured (there was not enough time to ask all questions in the interview guides and follow up all potentially interesting thoughts). This reinforces the argument above—that it may be a good idea to interview autistic people multiple times. This allows for deeper exploration of a topic while reducing risks of cognitive overload. Another idea could be to take a break after perhaps 40-45 minutes and then offer an additional interview session for those who have the cognitive capacity to continue. However, it is important to consider that this may not meet the needs of the autistic interviewer, as they need to remain sharp and focused the whole interview.

### Recommendations and considerations for autistic-friendly group interviewing

To summarize, in **Table 3** we provide an overview of our method of autistic interviewing that has been developed and explored throughout the paper. Several of the recommendations are in line with other neurodivergent researcher´s recommendations, such as Nicolaidis et al, (2, strengthening the relevance of them. We encourage readers to continue to revise and develop this method in their own research.

**Table 3:** Recommendations and considerations for autistic-friendly group interviewing.

Part of the research process	Actions
Research Team	• Autistic led
Research Design	• Consider how the design meets the needs of autistic people
Development of interview questions	• Try to use autistic-friendly language as much as possible (i.e, use words that are accepted and understood by the participants), or alternatively, phrase the questions openly enough to give participants space to find their own autistic language • Have follow-up questions that encourage the participant to provide examples • Possibilities of bottom-up translations of top-down questions (i.e. when questions originally designed from theoretical or conceptual frameworks (top-down) are reformulated starting from concrete experiences, specific details, or situated knowledge (bottom-up), thus allowing meaning to emerge through the participant’s own way of structuring understanding from the ground up). For example: instead of asking how they feel, ask what it feels like in the body in a certain situation, and build from there.
Participant Recruitment and preparation for the interview	• Clearly communicate the study’s objective and the flexibility in data collection • Be clear about how long the interview will last • Offer the participants the opportunity to read the interview questions beforehand
Conducting Interviews	• Led by an autistic interviewer • Clarify the goal of the study again (including level of abstraction and degree of specificity) • Emphasize that it is normal to feel uncertain at the beginning of a group interview and that warm-up time is needed to make participants feel more comfortable • Inform participants that their responses should not be framed as comparisons with neurotypicals • Accept autistic participants’ personal preferences to engage • Start by informing how the interview is “autistic-friendly” and why, when, and how the chat function can be used • Guide who should speak at the beginning before the conversation gets going • If the discussion drifts away from the main topic to subjects related to being in an autistic space, let it be for a while, as it fosters the group’s sense of community • Conduct several interviews with the same group
After the interviews	• Encourage participants to submit written post-interview reflections afterward
Data Quality & Integrity	• Autistic input on data analysis and interpretation • Provide autistic participants with opportunity to review their responses in the transcript and encourage participants to add post-interview reflections coming to their minds when reading the transcripts

## Conclusions

In this collective reflexive pilot study, we have provided examples of how an interview guide and an interview situation can be prepared, conducted, and evaluated in an autistic space regarding a personal topic (autistic flourishing) from a neurodiversity and biopsychosocial approach. This study highlights key considerations for conducting group interviews in autistic spaces, emphasizing the importance of clear communication and flexible interview structures. However, challenges such as the risk of questions being too broad or too top-down in framing remain. Future research should explore alternative formats, for example physical meetings, more narrow topics, using vignettes/cases, non-verbal data collection or repeated online group interviews to reach more depth. Given the exploratory nature of this study, the proposed approach to interviewing in autistic spaces warrants further testing in larger-scale studies to validate and expand upon these initial findings.

## Data Availability

The datasets presented in this article are not readily available because they contain sensitive personal reflections and contextual details that could compromise the author’s privacy and the ethical integrity of the autoethnographic research. Requests to access the datasets should be directed to asahed@hig.se.
